# Age-Related *Toxoplasma gondii* Seroprevalence in Dutch Wild Boar Inconsistent with Lifelong Persistence of Antibodies

**DOI:** 10.1371/journal.pone.0016240

**Published:** 2011-01-20

**Authors:** Marieke Opsteegh, Arno Swart, Manoj Fonville, Leo Dekkers, Joke van der Giessen

**Affiliations:** 1 Laboratory for Zoonoses and Environmental Microbiology, National Institute for Public Health and the Environment (RIVM), Bilthoven, The Netherlands; 2 Institute for Risk Assessment Sciences (IRAS), Faculty of Veterinary Medicine, Utrecht University, Utrecht, The Netherlands; 3 Animal Health Service (GD), Deventer, The Netherlands; Fundació Institut Germans Trias i Pujol; Universitat Autònoma de Barcelona CibeRES, Spain

## Abstract

*Toxoplasma gondii* is an important zoonotic pathogen that is best known as a cause of abortion or abnormalities in the newborn after primary infection during pregnancy. Our aim was to determine the prevalence of *T. gondii* in wild boar to investigate the possible role of their meat in human infection and to get an indication of the environmental contamination with *T. gondii*. The presence of anti-*T. gondii* antibodies was determined by in-house ELISA in 509 wild boar shot in 2002/2003 and 464 wild boar shot in 2007. Most of the boar originated from the “Roerstreek” (n = 673) or the “Veluwe” (n = 241). A binormal mixture model was fitted to the log-transformed optical density values for wild boar up to 20 months old to estimate the optimal cut-off value (−0.685) and accompanying sensitivity (90.6%) and specificity (93.6%). The overall seroprevalence was estimated at 24.4% (95% CI: 21.1–27.7%). The prevalence did not show variation between sampling years or regions, indicating a stable and homogeneous infection pressure from the environment. The relation between age and seroprevalence was studied in two stages. Firstly, seroprevalence by age group was determined by fitting the binary mixture model to 200 animals per age category. The prevalence showed a steep increase until approximately 10 months of age but stabilized at approximately 35% thereafter. Secondly, we fitted the age-dependent seroprevalence data to several SIR-type models, with seropositives as infected (I) and seronegatives as either susceptible (S) or resistant (R). A model with a recovery rate (SIS) was superior to a model without a recovery rate (SI). This finding is not consistent with the traditional view of lifelong persistence of *T. gondii* infections. The high seroprevalence suggests that eating undercooked wild boar meat may pose a risk of infection with *T. gondii*.

## Introduction

*Toxoplasma gondii* is an important zoonotic protozoan with a worldwide distribution that may cause abortion or abnormalities in the newborn. Cats are the definitive host of *T. gondii* and shed millions of oocysts into the environment after a primary infection. *T. gondii* infection is probably of limited clinical importance in wild boar: Severe clinical toxoplasmosis is considered rare in pigs [1], and although decreased reproductive performance was observed in *T. gondii* seronegative—and therefore at risk for primary infection—wild boar [2], there are no reports of clinical toxoplasmosis in wild boar. However, infected wild boar are a source of infection for people if their meat is eaten undercooked [3]. In addition, the prevalence in wild boar gives an indication of the environmental contamination, since they acquire their infection from contact with soil or by ingesting infected rodents or birds.

The *T. gondii* seroprevalence for the Dutch human population has decreased from 40.5% in 1995/1996 to 26.0% in 2006/2007 [4]. This is thought to be an effect of the decreased prevalence in consumption animals, especially in pigs, due to increased intensive indoor farming. A stable infection pressure from the environment is suggested by the unchanged seroprevalence in sheep when compared to studies in the eighties [5]. However, differences may have been missed due to methodological differences between studies, for example the cut-off value used in the serological assay or the number of confounders corrected for in the analysis. Therefore, we chose to compare the seroprevalence of *T. gondii* in wild boar for two years within the same study.

An in-house ELISA was used to test sera from 973 hunted wild boar originating from 2002/2003 and 2007. Because of a lack of appropriate reference sera a cut-off value was selected from a binormal mixture model fitted to the log-transformed optical density-values [5], and used to score wild boar positive or negative. Seroprevalence over sampling years and regions was subsequently compared by logistic regression analysis. The age-dependent seroprevalence was additionally estimated by fitting the mixture model per age category, and interpreted by fitting various compartmental infection models.

## Materials and Methods

### Study population and samples

Wild boar are omnivorous animals, although their diet consists mostly of vegetable matter [6]. They eat, for example, mast, roots, green plant matter, berries, and agricultural crops, but also fungi, earthworms, insects, eggs, small rodents and birds. Rooting behavior takes up much of their time resulting in intensive soil contact. In The Netherlands the breeding season starts around September, and between 1 and 11 piglets are born approximately 115 days later. In The Netherlands wild boar populations are tolerated in only two areas: 60 wild boar in the “Roerstreek” in the south on the border with Germany, and between 600 and 800 wild boar on the “Veluwe” in the centre of The Netherlands. In both areas the population is controlled by hunting, and in other areas all wild boar are shot. The landscape is similar in both areas, and is characterized by forest, moors and heath, pools and drift sand. Although *Felis silvestris* has been spotted incidentally in The Netherlands [7], [8] we assume that domestic and stray cats are the predominant source of oocysts in both areas.

Since 1994, serum samples of 60–80 animals randomly selected from the thousands of wild boar hunted on the Veluwe, and of all wild boar hunted in the Roerstreek are collected yearly at the Animal Health Service in Deventer. These sera are tested for antibodies against SVD-, PR-, FMD-, and CSF-virus and sent to the RIVM to test for antibodies against *Trichinella* [9]. At the RIVM these samples are stored at −20°C. Location, sex, and age in months as estimated from dental development are recorded. All samples available from the years 2002–2003 (n = 509) and 2007 (n = 464) were included in this study. Most samples originated from the “Roerstreek” (n = 673) and the “Veluwe” (n = 241). A small number of samples (n = 30) came from other areas, mostly from wild boar roaming up North from the Roerstreek, or across the German border into Gelderland and Overijssel.

### Serological assay

Sera were tested by in-house indirect ELISA and optical density (OD)-values corrected for plate-to-plate variation as described previously [5], but with the conjugate replaced by 1∶12.500 diluted polyclonal rabbit anti-swine HRP-labeled immunoglobulins (Dako, Heverlee, Belgium). The six control sera included on each plate varied in OD-value from 0.10 to 1.25.

### Data analyses

#### 1. Binormal mixture model to estimate cut-off

A binormal mixture model [5] was fitted to the log_10_-transformed corrected OD-values for wild boar up to 20 months of age (n = 722) to estimate the cut-off value at which the number of correctly scored animals is highest, and accompanying sensitivity and specificity. Analysis was limited to wild boar up to 20 months of age, as initial analysis including all wild boar did not satisfactorily fit the data.

#### 2. Logistic regression analysis to compare seroprevalence by region and year

The cut-off value obtained from the binormal mixture model was subsequently used to score wild boar as positive or negative. The apparent prevalence (*AP = pos/n*, where ‘*pos*’ is a random variable representing the number of positive animals in a sample of size *n* from the wild boar population) was calculated and corrected for sensitivity (*Se*) and specificity (*Sp*) using the Rogan-Gladen estimator, yielding the true prevalence (*TP = (AP+Sp−1)/J*, with *J = Se+Sp−1*) [10]. Confidence intervals were calculated using the normal approximation *TP±1.96 √var(TP)*, with *var(TP) = AP (1−AP)/nJ^2^* [11].

Seroprevalence was compared over years and regions. The relation between apparent prevalence and sex, age and sampling season was additionally studied as these factors are possible confounders. Animals were classified into 10 equal percentile age categories for univariable analysis, but age was included as a continuous variable in logistic regression analysis. Seroprevalence was checked univariably for differences among groups by Pearson's *χ* ^2^-test. Region, year, and all factors significant at the 85% confidence level, were included in logistic regression analysis (SPSS 18.0, SPSS Statistics, Chicago, IL, USA). Odds-ratios with 95% confidence intervals based on likelihood ratio statistics are reported.

#### 3. Mathematical model for relation between age and seroprevalence

Using the obtained binormal mixture model a direct estimate of the true seroprevalence per age category was obtained, making correction for sensitivity and specificity unnecessary. First the *n* animals were sorted by age. Then age groups of between 100 and 200 animals were composed according to *A(i) = {animals* max*(i−100,1) to* min*(i+99,n)}*, where *1<i<n*. For each group *A(i)* we determined the average age *a(i)*. Between every increase of *i*, animals of the same age were shuffled randomly in order to compensate for systematic bias. The seroprevalence *c(i)* for each age group *A(i)* was determined by fitting the binormal mixture model with means and standard deviations as obtained before, but leaving the mixing parameter to be estimated. For each group *A(i)* the estimated mixing parameter *c(i)*, i.e. the seroprevalence, was plotted against their mean age *a(i)*.

**Figure 1:**
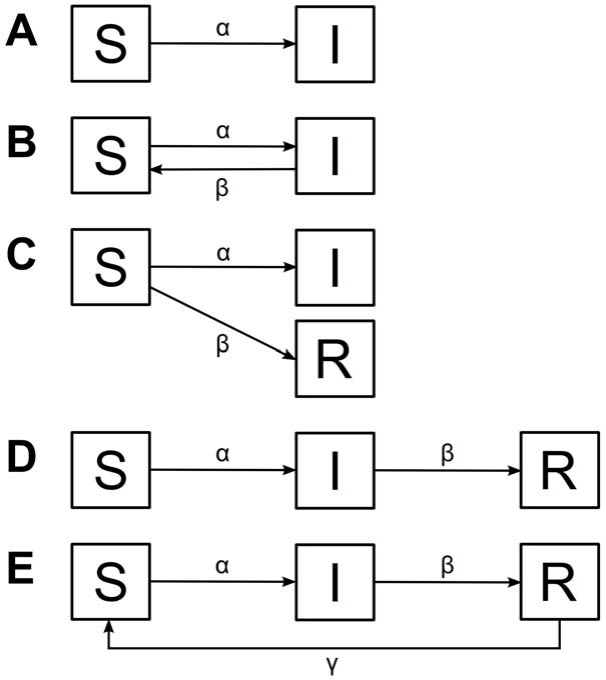
The compartmental infection models considered. S susceptible, I infected, R resistant. A) SI model: only infection, B) SIS model: reversion to susceptible possible, C) SI/SR model: either infection or resistance, D) SIR model: some time after infection resistance occurs, E) SIRS model: it is possible to lose resistance and regain susceptibility.

Various compartmental infection models (Fig. 1: SI, SIS, SI/SR, SIR, SIRS; with S being susceptibles, I being infecteds, i.e. seropositive animals, and R being animals resistant to infection without antibodies) were fitted against the observed age-seroprevalence data *(a(i),c(i))* using a least squares approach.

In steady state, the age- and time dependent dynamics and initial conditions for the SIR-model [12] are described by 
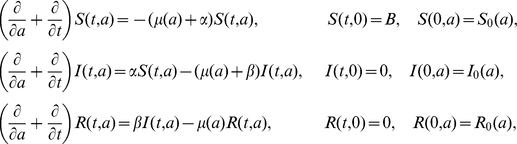



Here, the transition rates α (*S→I*) and β (*I→R*) are time and age independent, *B* is the number of births per month, and *μ(a)* is an age-dependent death rate. The quantities at time zero are considered known, but not needed later. We assume that the population is in steady state, i.e. every quantity is time-independent. Also, define *N = S+I+R* and set *s(a) = S(a)/N(a)*, *i(a) = I(a)/N(a)* and *r(a) = R(a)/N(a)*, then
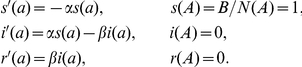



Note that the birth rates and death rates have conveniently dropped out of the equations. Also note that we have set the fraction of susceptibles equal to one at age *A*, the age at which newborns are first exposed to the environment (this involves the slight approximation *B = N(A)≈N(0)*). The solution to this system of equations for the seroprevalence *i(a)* is found to be
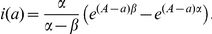



Transition rates *α* and *β* and age *A* at which seroprevalence was 0% were estimated using a least-squares fit to the seroprevalences grouped by age. Model fit was assessed by calculation of *R^2^* values. The other compartmental models were obtained by reduction of the described SIR-model (the fit to the SIRS model reduced to a SIS model).

## Results

### Cut-off and seroprevalence using binormal mixture model

**Figure 2:**
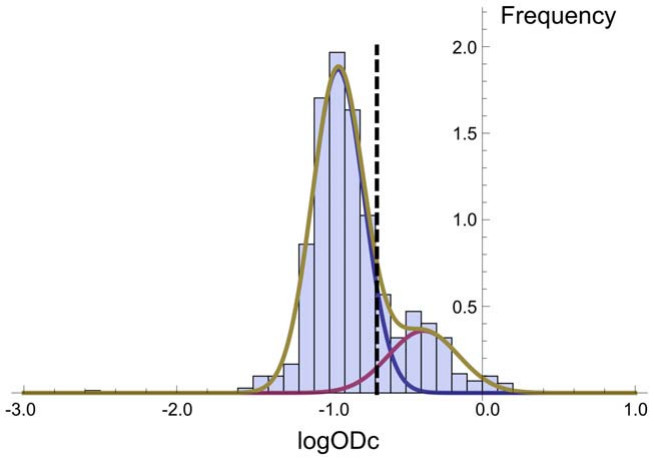
*T. gondii* ELISA results for wild boar. Frequency distribution of observed log_10_-transformed ODc-values in *T. gondii* ELISA for wild boar up to 20 months of age (n = 722) (bins), distributions fitted using the binormal mixture model (lines), and cut-off value (logODc = −0.685) (vertical dashed line).

After correction for plate-to-plate variation and log-transformation, the frequency distribution of optical density (OD) values was drawn. The observed distribution of log-transformed OD-values was best described assuming a mixture of two distributions (Fig. 2). Based on this mixture, the seroprevalence for the wild boar included (up to 20 months of age) was estimated at 20.5%. The cut-off value at which the number of animals scored correctly is maximal was estimated at −0.685, with an AUC for the ROC-curve of 0.975, a sensitivity of 90.6% and a specificity of 93.6%.

### Seroprevalence and risk factors using logistic regression analysis

**Figure 3:**
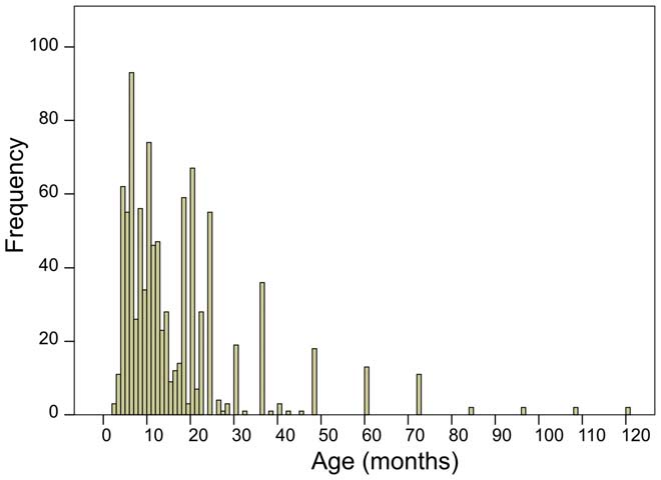
Frequency distribution of estimated age in months for 932 wild boar.

Estimated age was available for 932 animals (Fig. 3), region of origin and sampling season for 944, and sex for 762 animals. For all wild boar tested (n = 973) the apparent prevalence was 26.9% and true prevalence 24.4% (95% CI: 21.2–27.7%). Univariable analysis showed significant differences in seroprevalence by age category (Table 1, p<0.0005), and a significant relation with region and sampling season at the 85% confidence level (Table 2). Sampling season was not significant in the logistic regression analysis (p = 0.053) and left out of the final model. The final model with region, year and age including all wild boar showed a significant increase in seroprevalence with age in months (OR 1.02, 95% CI: 1.01–1.03), but no regional or temporal differences (Table 3). The model fitted the data (Hosmer and Lemeshow test [13], p = 0.068).

**Table 1:** Wild boar serum samples positive for antibodies against *T. gondii* and mean age (months) by age category.

Age category	Mean age	Positive (%)	Total
≤5	4.3	13 (9.9)	131
6	6.0	17 (18.3)	93
7–8	7.7	24 (29.3)	82
9–10	9.7	32 (29.6)	108
11–12	11.5	24 (25.8)	93
13–15	13.8	13 (21.7)	60
16–19	17.6	26 (29.5)	88
20–22	20.6	30 (29.4)	102
23–30	25.7	38 (46.3)	82
≥31	51.9	30 (32.2)	93
total	16.4	247 (26.5)	932

**Table 2:** Wild boar serum samples positive for antibodies against *T. gondii* by sampling year, region, sampling season and sex; and p-values for Pearson's χ^2^-statistic per variable.

Variable	Category	Positive (%)	Total	p-value
Sampling year	2002/2003	134 (26.3)	509	
	2007	128 (27.6)	464	0.658
Region	Roerstreek	171 (25.4)	673	
	Veluwe	67 (27.8)	241	
	other	15 (50.0)	30	0.011
Sampling season	winter	54 (24.7)	219	
	spring	17 (22.4)	76	
	summer	75 (24.4)	308	
	autumn	107 (31.4)	341	0.119
Sex	male	113 (28.7)	394	
	female	100 (27.2)	368	0.350

**Table 3:** Odds ratios for sampling year, region and age in months as risk factors for *T. gondii* seroprevalence in wild boar (n = 932).

Variable	Category	OR	95% CI	p-value
Sampling year	2002/2003	reference		
	2007	1.07	0.79–1.45	0.647
Region	Roerstreek	reference		
	Veluwe	1.05	0.75–1.49	0.768
	other	2.33	0.92–5.89	0.074
Age (months)		1.02	1.01–1.03	0.001

### Age-seroprevalence relation

**Figure 4:**
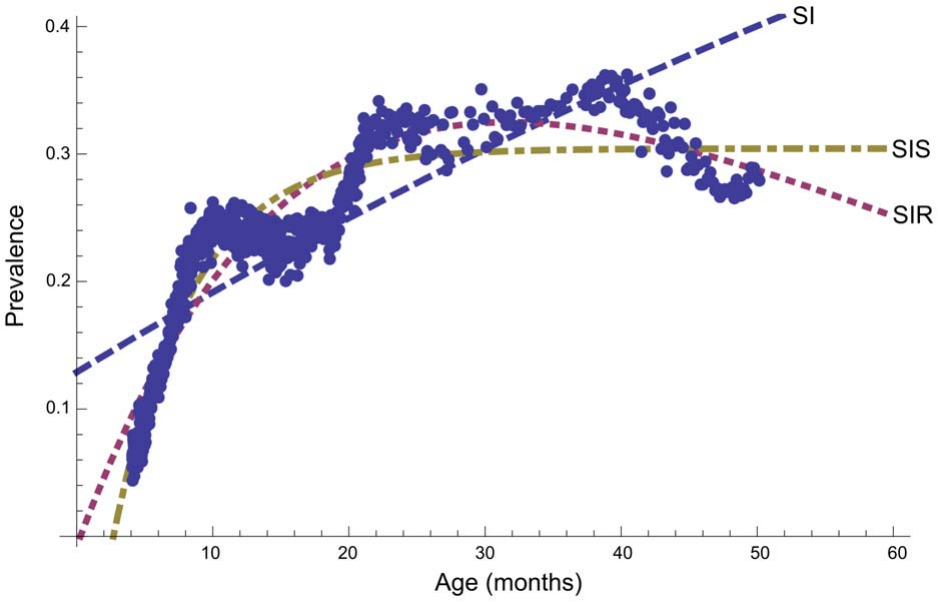
*T. gondii* seroprevalence in wild boar by age. Seroprevalence estimated by fitting the binormal mixture model to age groups including 100–200 animals plotted at the mean age of the group (dots); and predicted seroprevalence by age using SI, SIR and SIS compartmental infection models (lines).

The seroprevalence by mean age per age group showed a steep increase at first, but seemed to stabilize at approximately 35% at 10 months of age (Fig. 4). The fitted compartmental models are shown in Figure 4 and the estimated transition rates, age with prevalence 0%, and model fit in Table 4. The SIRS model is not shown; the fit yielded γ = 1, and other parameters the same as the SIS model. Then, the expression for the prevalence is the same as that for the SIS model. Also, parameter estimates for the SI/SR were such that again we obtained equivalency to the SIS model. The SIS model, in which animals can revert to susceptible state, fitted the data best (*R* ^2^-value of 0.88, Table 4). Using this model the incidence rate is estimated at 0.050 per month, while each month 0.11 of infected animals become susceptible again. This results in an average time being seropositive of 9 months. Using this model, the seroprevalence is estimated at 0% until 2.5 months of age.

**Table 4:** Transition rates (α,β), age in months with seroprevalence 0% (A), average time in preceding state in months (T), model fit (*R^2^*), and prevalence equation for SIR, SI, and SIS-model fitted to the age-related *T. gondii* seroprevalence in 932 wild boar.

Model	Parameter	Value	T	*R* ^2^	Prevalence
SIR	Rate of infection	α = 2.8e−2	36	0.85	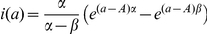
	Rate of resistance	β = 3.5e−2	29		
	Age with prevalence 0%	A = 2.3e−1			
SI	Rate of infection	α = 7.6e−3	131	0.63	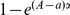
	Age with prevalence 0%	A = −17.6	-		
SIS	Rate of infection	α = 5.0e−2	20	0.88	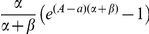
	Rate of reversion	β = 1.1e−1	9		
	Age with prevalence 0%	A = 2.5			

## Discussion

It was our aim to study the seroprevalence in wild boar and study temporal and regional variations therein. We used our in-house ELISA to test sera and selected a cut-off value with accompanying test characteristics by binormal mixture analysis. Logistic regression analysis showed that the prevalences found did not differ significantly over the sampling years or between regions. This indicates a stable and homogeneous infection pressure from the environment. The observed seroprevalence in wild boar (24.4%) is much higher than the seroprevalence of approximately 3% in Dutch outdoor-reared pigs [14], [15], but is much lower than the seroprevalence of almost 60% in fattening pigs in The Netherlands in the sixties [16], and is comparable to the seroprevalence in wild boar from Champagne-Ardenne [17], Czech republic [18], and Austria [19].

Using the binormal mixture model, estimates of true prevalence were obtained directly for the different age groups. Plotting these seroprevalences against the mean ages of the groups showed a steep increase in seroprevalence up to 10 months, but a stable situation thereafter. *T. gondii* infection is generally believed to persist lifelong in most hosts [20], and stable OD-values and persistence of tissue cysts has been demonstrated in pigs up to 1 to 2 years post experimental infection [21]. However, the SI-model, that assumes a constant incidence rate among susceptibles and lifelong immunity, did not fit the observed seroprevalence by age curve well. The SIS-model, that includes reversion to susceptible after infection, fitted the data much better. This suggests a loss of antibodies that may have been preceded by a loss of tissue cysts. However, a model with the same fit was also obtained by moving animals from susceptible into either infected or resistant (SI/SR-model), and could probably also arise by incorporating age-dependent parameters, or a combination of these effects. We prefer the SIS model over the SI/SR or SIR model, for the reason that the ‘R’ compartment is hard to interpret: The animal is supposed to be immune without presence of antibodies. In addition, the rate at which resistant animals become susceptible again is estimated at one in the SIRS-model, which rejects the hypothesis of temporary resistance to infection after antibodies have waned. Including age-dependent parameters in the model (of unknown age dependency!) adds to the complexity – a simpler model with good explanatory power may be preferred. Temporal variation in infection pressure from the environment can also influence the age-prevalence relation: For example, the prevalence in older people may be higher as they have consumed meat from animals with a high prevalence from before animal husbandry was industrialized. However, as the seroprevalence was shown stable over sampling years that are further apart than the life-expectancy of wild boar, such an effect is unlikely here, and therefore the use of compartmental infection models that inherently assume a constant infection pressure is appropriate. Observations inconsistent with lifelong persistence have been reported previously: Several authors report that no statistically significant effect of age on seroprevalence of *T. gondii* in wild boar was observed [22], [23], [24], [25], [26], whereas only one study did find a significantly higher prevalence in adult wild boar [2]. In addition, tissue cysts were detected by mouse bioassay in the heart of only 50% of 20 seropositive (MAT titer ≥1∶24) wild boar in France [17], and although paired results per wild boar are not presented, the prevalence of *T. gondii* by bioassay (2%) was much lower than by Sabin Feldman Dye test (15%) in a Czech study [27].

Seroprevalence could only be calculated for animals of at least 5 months of age as not enough younger boar were sampled. There are two reasons why the predicted age-prevalence relation cannot be extrapolated to younger ages. Firstly, abandoning the nest and weaning occur gradually [28], [29]. Therefore, exposure to environmental oocysts and, consequently, the infection rate are still increasing at very young age. Secondly, young piglets may be protected against infection by maternal antibodies, leading to a measurable but transient antibody titer that protects them against infection but is not the result of infection. As weaning of the entire litter is complete at on average 17.2 weeks after birth for domestic pigs in a semi-natural environment [29] both an estimated seroprevalence of 0% until 2.5 months of age (SIS-model), and a seroprevalence of 13% at age 0 (SI-model) due to maternal antibodies are possible, but, as explained, the actual seroprevalences may differ.

In conclusion, we have shown a high seroprevalence of *T. gondii* in wild boar that was equal over the sampling years and regions. As the seroprevalence is high, consumption of raw or undercooked wild boar meat may pose an important risk of infection. The stable seroprevalence indicates a constant infection pressure from the environment. In addition, we found an age-seroprevalence relation that is inconsistent with a constant infection rate in combination with lifelong immunity. A model including reversion to susceptible state fitted the data nicely. This may mean that a negative serological test does not exclude prior exposure to *T. gondii* and, if the loss of antibodies is preceded by a loss of tissue cysts, that wild boar can clear their infection. But before drawing these conclusions, the actual mechanism behind the stabilization of seroprevalence at around 35% requires further investigation, for example by longitudinal follow-up of infected wild boar regarding presence of antibodies and tissue cysts.
